# Correction: Subdomains of the *Helicobacter pylori* Cag T4SS outer membrane core complex exhibit structural independence

**DOI:** 10.26508/lsa.202402801

**Published:** 2024-05-13

**Authors:** Jacquelyn R Roberts, Sirena C Tran, Arwen E Frick-Cheng, Kaeli N Bryant, Chiamaka D Okoye, W Hayes McDonald, Timothy L Cover, Melanie D Ohi

**Affiliations:** 1 Life Sciences Institute, University of Michigan , Ann Arbor, MI, USA; 2 Department of Biological Chemistry, University of Michigan , Ann Arbor, MI, USA; 3 Department of Medicine, Vanderbilt University School of Medicine, Nashville, TN, USA; 4 Department of Pathology, Microbiology and Immunology, Vanderbilt University School of Medicine, Nashville, TN, USA; 5 Proteomics Laboratory, Mass Spectrometry Research Center, Vanderbilt University School of Medicine, Nashville, TN, USA; 6 Veterans Affairs Tennessee Valley Healthcare System, Nashville, TN, USA; 7 Department of Cell and Developmental Biology, University of Michigan , Ann Arbor, MI, USA

## Abstract

Structural and proteomic analysis of *H. pylori* Cag T4SSs purified from deletion mutants highlight the unexpected structural independence between the OMC and PR, two major subdomains of this complex.

Article: Roberts JR, Tran SC, Frick-Cheng AE, Bryant KN, Okoye CD, McDonald WH, Cover TL, Ohi MD (2024 April 17) Subdomains of the *Helicobacter pylori* Cag T4SS outer membrane core complex exhibit structural independence. Life Sci Alliance 7(6): e202302560. doi: 10.26508/lsa.202302560. PMID: 38631913.

In this manuscript, there was an error in the Data Availability section where the ∆*cagT* PR EM map was incorrectly uploaded and referenced as “EMD-42395.” The correct EM map has now been uploaded and the map has a new accession number, “EMD-44587.”

## Supplementary Material

Reviewer comments

