# Supplementary material for: Subdomains of the Helicobacter pylori Cag T4SS outer membrane core complex exhibit structural independence
Source: Life Sci Alliance. 2024 Apr 17;7(6):e202302560. doi: 10.26508/lsa.202302560 (PMC11024343; doi:10.26508/lsa.202302560)
Supplement: Supplementary file 1 [file LSA-2023-02560_TableS1.docx]

**Supplementary Table 1: Cryo-EM data collection, refinement, and statistics**

|  | CagY∆AP OMC  EMD-42290 | | | | CagY∆AP PR  EMD-42392 | | Δ*cagT* PR  EMD-42395 | *∆cagM* PR  EMD-42393 |
| --- | --- | --- | --- | --- | --- | --- | --- | --- |
| **Data Collection and Processing** | |  |  |  | |  |  |  |
| Magnification | 81,000X | | | | 81,000X | | 81,000X | 81,000X |
| Voltage (kV) | 300 | | | | 300 | | 300 | 300 |
| Bioquantum slit width (eV) | 20 | | | | 20 | | 20 | 20 |
| Electron exposure (e^-^/Å^2^) | 60 | | | | 60 | | 60 | 60 |
| Defocus range (µm) | -1.0 - -2.5 | | | | -1.0 - -2.5 | | -1.0 - -2.5 | -1.0 - -2.5 |
| Pixel Size (Å) | 1.08 | | | | 1.08 | | 1.08 | 1.08 |
| Symmetry imposed | C14 | | | | C17 | | C17 | C17 |
| Initial particle images (no.) | 22,643 | | | | 22,643 | | 8,415 | 4,251 |
| Final particle images (no.) | 22,643 | | | | 22,643 | | 5,706 | 3,009 |
| Map resolution (Å) | 3.8 | | | | 6.6 | | 8.0 | 8.5 |
| FSC threshold | 0.143 | | | | 0.143 | | 0.143 | 0.500 |
| Map resolution range (Å) | 2-8 | | | | 6-12 | | 7-12 | 4-10 |
