# Supplementary material for: Correction: Subdomains of the Helicobacter pylori Cag T4SS outer membrane core complex exhibit structural independence
Source: Life Sci Alliance. 2024 May 13;7(8):e202402801. doi: 10.26508/lsa.202402801 (PMC11091449; doi:10.26508/lsa.202402801)
Supplement: Reviewer comments [file LSA-2024-02801_review_history.pdf]

Life Science Alliance

Correction: Subdomains of the Helicobacter pylori Cag T4SS outer membrane core complex exhibit structural independence

Jacquelyn Roberts, Sirena Tran, Arwen Frick-Cheng, Kaeli Bryant, Chiamaka Okoye, W. McDonald, Timothy Cover, and Melanie Ohi
DOI: https://doi.org/10.26508/lsa.202402801

Corresponding author(s): Melanie Ohi, University of Michigan-Ann Arbor

Review Timeline:

Submission Date:	2024-04-30
Accepted:	2024-05-07

Scientific Editor: Eric Sawey, PhD

Transaction Report:

(Note: With the exception of the correction of typographical or spelling errors that could be a source of ambiguity, letters and reports are not edited. The original formatting of letters and referee reports may not be reflected in this compilation.)

May 7, 2024

RE: Life Science Alliance Manuscript #LSA-2024-02801

Dr. Melanie D Ohi
University of Michigan-Ann Arbor
Life Sciences Institute
210 Washtenaw Avenue
Ann Arbor, Michigan 48109

Dear Dr. Ohi,

Thank you for submitting your Correction entitled "Correction: Subdomains of the Helicobacter pylori Cag T4SS outer membrane core complex exhibit structural independence".

IMPORTANT: If you will be unreachable at any time, please provide us with the email address of an alternate author. Failure to respond to routine queries may lead to unavoidable delays in publication.

You can contact the journal office with any questions, contact@life-science-alliance.org

Sincerely,

Eric Sawey, PhD
Executive Editor
Life Science Alliance
http://www.lsajournal.org

May 7, 2024

RE: Life Science Alliance Manuscript #LSA-2024-02801

Dr. Melanie D Ohi
University of Michigan-Ann Arbor
Life Sciences Institute
210 Washtenaw Avenue
Ann Arbor, Michigan 48109

Dear Dr. Ohi,

Thank you for submitting your Correction entitled "Correction: Subdomains of the Helicobacter pylori Cag T4SS outer membrane core complex exhibit structural independence".

IMPORTANT: If you will be unreachable at any time, please provide us with the email address of an alternate author. Failure to respond to routine queries may lead to unavoidable delays in publication.

You can contact the journal office with any questions, contact@life-science-alliance.org

Sincerely,

Eric Sawey, PhD
Executive Editor
Life Science Alliance
http://www.lsajournal.org
